# Granulomatous myocarditis arising from intravesical Bacillus Calmette–Guérin therapy leading to death diagnosed by postmortem examination: a case report

**DOI:** 10.1186/s13256-023-04310-4

**Published:** 2024-01-10

**Authors:** Saroja D. Geetha, Hector D. Chavarria, Mohammed Abdelwahed, Nidhi Kataria, Vanesa Bijol, Kasturi Das

**Affiliations:** Northwell Health, Department of Pathology, Zucker School of Medicine, North Shore University Hospital/Long Island Jewish Medical Center, 2200 Northern Blvd, Suite 104, Greenvale, NY 11548 USA

**Keywords:** Cardiac granuloma, Granulomatous myocarditis, Arrythmia, Urothelial cancer, Bladder cancer, BCG therapy, Intravesical BCG, Systemic BCG-osis, Multiorgan granulomas, Death

## Abstract

**Background:**

Intravesical Bacillus Calmette–Guérin (BCG) is used as a standard adjuvant therapy for non-muscle invasive urothelial cancer. Most patients tolerate the treatment well, with mild side effects. Systemic complications are extremely rare, occur due to BCG dissemination and are associated with immunocompromised state and urothelial breach.

**Case presentation:**

We present a case of a 78-year-old male, a former smoker, with history of non-muscle invasive urothelial carcinoma status post partial resection followed by intravesical BCG therapy. An autopsy was performed due to the sudden nature of his death. Autopsy showed multiple necrotizing granulomas in the brain, atrium, ventricles, lungs, kidneys, and urinary bladder. Stains for acid-fast bacilli and fungi were negative. In addition, bilateral lungs showed evidence of bronchopneumonia secondary to cytomegalovirus.

**Conclusion:**

Granulomatous myocarditis arising from BCG therapy is extremely rare. Our patient with urothelial cancer treated with BCG developed multiorgan granulomas, most likely due to a hypersensitivity reaction to intravesical BCG. Arrhythmia induced by granulomatous myocarditis was the cause of his death. Although there have been few cases of systemic BCG-osis causing fatal sepsis leading to death, a cardiac cause of death is unique.

## Background

Bacillus Calmette–Guérin (BCG) is the live attenuated form of *Mycobacterium bovis* used as a vaccine for protection against tuberculosis and non-tuberculous mycobacterial infection. Later, intravesical BCG installation became one of the treatment options for non-muscle invasive bladder cancer. This therapy is associated with a variety of adverse events, most of which are tolerable or controllable with supportive care. Few patients suffer severe side effects resulting from disseminated BCG infection, such as hepatitis, prostatitis, pneumonitis, arthritis, and mycotic pseudoaneurysms. Sepsis with fatal outcomes have been reported but are rare. Here we report a unique case of systemic BCG-osis with development of cardiac granulomas causing death.

## Case presentation

**Fig. 1:**
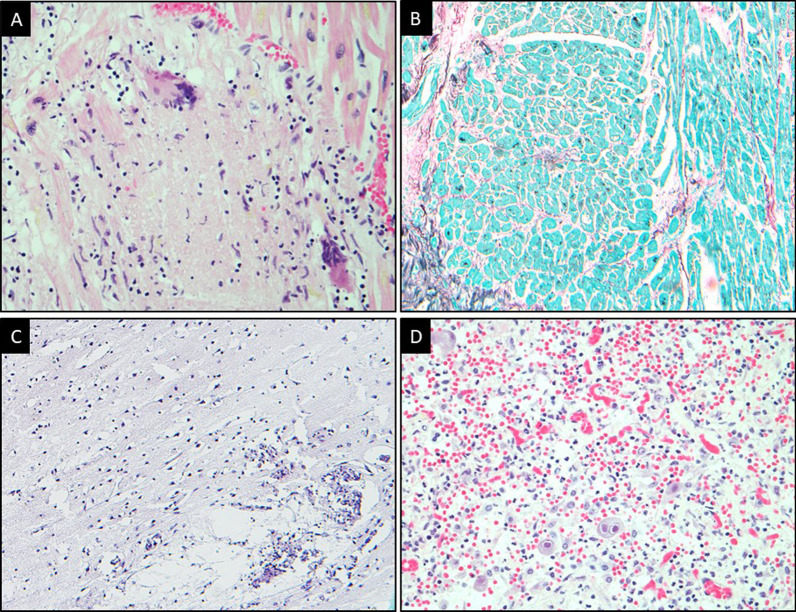
**A** Granulomatous inflammation of the myocardium with multinucleate giant cells (H&E, 40×). **B** Absence of acid bacilli in the myocardial tissue (AFB stain, 10×), **C** Cerebral tissue with microglial nodules (H&E, 10×), **D** Lung tissue exhibiting pneumonia with cells showing prominent basophilic nuclear inclusions and clear halo consistent with CMV effect (H&E, 40×) are shown

We present a case of a 78-year-old man, a former smoker, with multiple chronic medical conditions on whom an autopsy was performed due to the sudden nature of his death. His cardiovascular history was significant for coronary artery disease, atrial fibrillation, and aortic stenosis status post-aortic valve replacement. A year prior, he had been diagnosed with non-invasive urothelial carcinoma and had undergone partial bladder resection followed by BCG therapy. His cancer was in remission. On follow up visits, he was found to have on and off fevers, following thorough multi-disciplinary investigations it was deemed to be a fever of unknown origin. One fine day, he collapsed at home and died. The sudden nature of his death, prompted a post-mortem examination. No specific gross findings were identified. However, microscopic examination revealed scattered necrotizing granulomas in the heart (Fig. 1A), both atria and ventricles, kidneys, bladder, and brain (Fig. 1C). Lungs showed non-necrotizing granulomas with bilateral bronchopneumonia (Fig. 1D) secondary to cytomegalovirus (CMV) infection as confirmed by CMV staining. The granulomas were negative for acid-fast bacilli (AFB) (Fig. 1B) and Grocott’s methenamine silver (GMS) stain.

## Discussion and conclusions

Bladder cancer is the sixth most common cancer in the United States of America [1]. Incidence of bladder cancer increases with age, with the median age of diagnosis being 73 years [2]. The most common presenting symptom is painless hematuria. Histologically, bladder tumors can be classified as urothelial and non-urothelial. Urothelial cancer, also known as transitional cancers, are the most common type, accounting for 90% of all bladder cancers [3]. Management options are dependent on the depth of tumor invasion [3]. For muscle invasive tumors, radical cystectomy and chemotherapy is the mainstay. For superficial tumors, which includes carcinoma *in situ*, and non-muscle invasive tumors, transurethral resection of the bladder tumor followed by intravesical chemotherapy or BCG are the treatment options.

BCG primarily used as a vaccine for protection against severe and disseminated tuberculosis infection was discovered by bacteriologist–veterinarian duo, Albert Calmette and Camille Guérin. It was found that development of bacterial infections in patients with lymphosarcoma was known to cause disease remission, which resulted in the use of bacterial extracts as adjuvant therapy for head and neck tumors [4]. Further studies showed that patients with active tuberculosis infections were less likely to develop cancer and that cancer survivors had a higher incidence of tuberculosis infection compared with those who succumbed to cancer [5]. Soon studies were published demonstrating the usage of BCG in treatment of systemic lymphoblastic leukemias, primary cutaneous melanomas, and metastatic melanomas [6, 7]. Treatment efficacy was observed to be better with small, localized tumors, and with tumors that had direct contact with BCG. Based on these findings, Morales et al. [8] used intravesical BCG as adjuvant therapy for superficial urothelial carcinomas, which was a major success. Theories behind its anticancer effects are still evolving and the most studied include antiproliferative and cytotoxic effects on tumor cells [9, 10].

As with every immunotherapy, use of BCG is associated with side effects. Fever following BCG installation is the most common. Although it is indicative of good immune activation, it may also be the initial sign of sepsis. Allergic reactions include arthralgias, cramps, and rash. Local side effects are noted in majority of patients and result due to direct irritation of the urogenital lining. These include chemical cystitis, bladder contracture, epididymitis, prostate abscess, and urethral strictures [11–13]. Systemic complications occur due to BCG dissemination and are rare. It can be seen in immunocompromised individuals such as pregnant women, patients with diabetes, hematologic malignancies, or acquired immunodeficiency syndrome (AIDS). Another risk factor associated with dissemination is a breach in the urothelium, which promotes hematogenous spread and is commonly seen in the setting of catheterization [14]. Systemic complications include spondylodiscitis, mycotic pseudoaneurysm, pneumonitis, peritonitis, and granulomatous inflammation involving kidneys, prostate, liver, and lymph nodes [13, 15–17]. However, viable bacteria are not always identified. In such cases, a type four hypersensitivity reaction to BCG has been postulated [18, 19]. Granulomatous cardiomyopathy is a rare entity and can be seen in tuberculosis infection. They are known to cause ventricular tachycardia and arrhythmias [20]. To our best knowledge, BCG therapy causing cardiac granulomas is extremely rare and has not been reported before.

We present a BCG-treated patient with urothelial cancer with postmortem findings of multiple granulomas in the brain, heart, lungs, bladder, and kidneys, most likely due to systemic BCG-osis. AFB stain was negative for viable microorganisms favoring the hypersensitivity reaction rather than direct infection. An immunodeficiency state demonstrated by CMV pulmonary infection could have been a predisposing factor. Arrythmias induced by granulomatous myocarditis was the cause of his death. Although there have been few cases of systemic BCG-osis causing fatal sepsis leading to death, a cardiac cause of death from this complication is unique.

## Data Availability

Not applicable.

## Abbreviations

BCG: Bacillus Calmette-Guérin
CMV: Cytomegalovirus
